# Characterization and Performance of Lactate-Feeding Consortia for Reductive Dechlorination of Trichloroethene

**DOI:** 10.3390/microorganisms9040751

**Published:** 2021-04-02

**Authors:** Jiangwei Li, Anyi Hu, Shijie Bai, Xiaoyong Yang, Qian Sun, Xu Liao, Chang-Ping Yu

**Affiliations:** 1 CAS Key Laboratory of Urban Pollutant Conversion, Fujian Key Laboratory of Watershed Ecology, Institute of Urban Environment, Chinese Academy of Sciences, Xiamen 361021, China; jwli@iue.ac.cn (J.L.); ayhu@iue.ac.cn (A.H.); xyan@env.dtu.dk (X.Y.); qsun@iue.ac.cn (Q.S.); xliao@iue.ac.cn (X.L.); 2 University of Chinese Academy of Sciences, Beijing 100049, China; 3 Institute of Deep Sea Science and Engineering, Chinese Academic of Sciences, Sanya 572000, China; baishijie@idsse.ac.cn; 4 Water Innovation, Low Carbon and Environmental Sustainability Research Center, National Taiwan University, Taipei 10617, Taiwan

**Keywords:** biostimulation, dechlorination, trichloroethene, lactate, microbial community, assembly mechanism, co-occurrence network

## Abstract

Understanding the underlying mechanism that drives the microbial community mediated by substrates is crucial to enhance the biostimulation in trichloroethene (TCE)-contaminated sites. Here, we investigated the performance of stable TCE-dechlorinating consortia by monitoring the variations in TCE-related metabolites and explored their underlying assembly mechanisms using 16S rDNA amplicon sequencing and bioinformatics analyses. The monitoring results indicated that three stable TCE-dechlorinating consortia were successfully enriched by lactate-containing anaerobic media. The statistical analysis results demonstrated that the microbial communities of the enrichment cultures changed along with time and were distinguished by their sample sources. The deterministic and stochastic processes were simultaneously responsible for shaping the TCE-dechlorinating community assembly. The indicator patterns shifted with the exhaustion of the carbon source and the pollutants, and the *tceA*-carrying *Dehalococcoides,* as an indicator for the final stage samples, responded positively to TCE removal during the incubation period. *Pseudomonas*, *Desulforhabdus*, *Desulfovibrio* and *Methanofollis* were identified as keystone populations in the TCE-dechlorinating process by co-occurrence network analysis. The results of this study indicate that lactate can be an effective substrate for stimulated bioremediation of TCE-contaminated sites, and the reduction of the stochastic forces or enhancement of the deterministic interventions may promote more effective biostimulation.

## 1. Introduction

Chloroethenes, such as trichloroethene (TCE), are halogenated aliphatic organic compounds that have been widely used as ingredients in industrial cleaning solutions and as “universal” degreasing agents due to their unique properties and solvent effects [1]. Due to its improper usage and indiscriminate disposal, TCE has been frequently detected with its concentration above 10 parts per billion [2], and has severely accumulated in certain anaerobic subsurface environments [3] and is often emitted into the atmosphere in vapor form, stripped either from groundwater or wastewater treatment plants (WWTPs) [4,5], which poses a serious risk to human health and can result in the generation of vinyl chloride (VC), a known human carcinogen [6], via natural or induced reduction processes [1,7].

Due to the persistent nature of TCE in the environment, the development of effective and economical methods for its remediation has become a prime concern for researchers, and various physical and chemical methods have been developed and applied for TCE removal from contaminated environmental components [8]. With the advantages of cost-effectiveness, high-throughput, and an ecofriendly nature, bioremediation is an attractive approach for treating TCE-contaminated sites compared with physicochemical processes [8]. The bioremediation of TCE in contaminated sites can occur through natural attenuation or through treatment via biostimulation or bioaugmentation. Even though natural dehalohydrolysis of TCE is possible, the half-lives of such reactions are in the order of days to centuries, and therefore, natural dehalohydrolysis is not considered as a significant mechanism of degradation [1]. Furthermore, traditional wastewater or municipal water supply treatment systems that utilize coagulation, sedimentation, precipitative softening, filtration, and chlorination have proven to be ineffective in reducing the concentrations of TCE to nonhazardous levels [9]. Below the water table, TCE can undergo anaerobic dechlorination under favorable geochemical and microbiological conditions, but the biodegradation rates are highly dependent on the site conditions and on engineering intervention [10]. Biostimulation is used to create subsurface conditions in which naturally occurring TCE-degrading bacteria thrive and grow, resulting in enhanced dechlorination of TCE. This in situ anaerobic treatment technology can help prevent the transportation of contaminants to the surface, thereby reducing the potential risk to humans, and has thus become a more favorable approach.

Under anaerobic conditions, TCE is degraded via dichloroethene (DCE) and VC to ethene (Eth) and to ethane via anaerobic reductive dechlorination, with hydrogen (H_2_) generated from organic substrates as the electron donor [11]. Reductive dechlorination is an electron-consuming process and is often limited by the availability of suitable electron donors in contaminated environments [12]. In the field of in situ bioremediation, fermentable substrates such as ethanol, acetate, lactate, gluconate, propionate, and butyrate, or complex organic materials, such as cane molasses, vegetable oil, surfactants, flour, nonfat milk, whey, corn cobs, wood chips, newsprint, and municipal waste sludge, were introduced to aquifers to stimulate the growth of indigenous or inoculated dechlorinators [10,11,12,13,14,15]. The reductive dechlorination of chlorinated solvents has also been observed in relation to a wide variety of methanogenic and nonmethanogenic consortia with various bacterial genera, such as *Clostridium*, *Dehalobacter*, *Dehalococcoides* (DHC), *Desulfitobacterium*, *Desulfuromonas*, *Dehalospirillum*, *Geobacter,* and *Sulfurospirillum*, were found to degrade chloroethenes [12,16,17]. However, only DHCs are capable of the complete reductive dechlorination of TCE to the benign product ethene, which means DCE or VC, or both, can be accumulated if DHC is absent in contaminated sites [16,18]. DHCs are strict hydrogenotrophs characterized by specific requirements for an exogenous supply of key compounds, including H_2_, acetate, corrinoids, biotin, and thiamine, all of which are generally provided by other microbial members of anaerobic food webs within dechlorinating communities [19].

The enrichment culture presents a complicated mixed-culture system, which involves competition, cooperation, or coordination among the bacterial communities [20]. The successful enrichment of TCE-dechlorinating consortia depends, to a great extent, on the appropriate stimulating conditions, which can promote the effective cooperation between the DHC and coexisting members through the exchange of substrates. Therefore, detailed investigations into the cooperative relationship between functional populations and the assembly mechanisms of key ecological members will contribute to the development of effective enriching strategies for the bioremediation of TCE-contaminated sites. Co-occurrence network analysis, as a practical tool, was employed to explore the interspecies association within the complex microbial assemblages in natural and anthropogenic environments [21], and their findings implied that the biotic interactions govern the community assembly and ecosystem function, and some central or keystone species play an essential role in maintaining the structure and function of the whole microbial community [22,23]. The exploration of the ecological succession can help us to understand the microbial community assembly mechanisms, and many previous studies have demonstrated that microbial community assembly is shaped by deterministic (e.g., environmental selection and biotic interactions) and stochastic (e.g., birth, death, speciation and dispersal events) processes simultaneously [24,25]. As a rapidly fermented substrate, lactate provides a more rapid release of electrons with corresponding high H_2_ partial pressures, and has been successfully used to enrich TCE-dechlorinating consortia [11,26,27,28,29]. However, at present, the assembly mechanisms of key ecological members and the microbial community succession patterns during the enriching process with lactate-feeding remain poorly understood. 

In this study, we aimed to address the performance of stable TCE-dechlorinating consortia with lactate-feeding and obtain a more thorough understanding the ecological mechanisms controlling microbial community assembly and interspecies interactions during the whole enriching processes. To assess TCE dechlorination, three stable TCE-dechlorinating enrichment cultures from different sources were established with TCE supplementation and lactate-feeding. Afterwards, we employed 16S rRNA gene amplicon sequencing and quantitative polymerase chain reaction (qPCR) analysis for reductive dehalogenase (RDase) genes from various time-series samples to track the dynamic variations in the microbial communities, their interactive networks, and the structure and function of microbiomes in response to long-term subculturing processes. Overall, this study provides a fundamental understanding of the enriching mechanism in the biostimulation process and is expected to facilitate the development of bioremediation strategies for TCE-contaminated sites.

## 2. Materials and Methods

### 2.1. Enrichment of TCE Dechlorinating Cultures

The TCE-dechlorinating culture was enriched using an anaerobic medium containing salts, trace elements, and vitamins and was prepared as previously described [30,31,32,33]. Two soil samples were collected from 3.5–5.5 m below ground level at different chlorinated solvent-contaminated sites (CS1 and CS2) in Shanghai as well as one activated sludge (AS) sample from a WWTP in Xiamen, China. All experiments were carried out in 100-mL serum bottles containing 40 mL (final volume) of the growth medium, which were sealed with black butyl rubber septa and aluminum crimp caps. In brief, a 4-g sample was added to 36 mL of the anaerobic medium and was amended with 250-μM TCE as the electron acceptor and 10-mM lactate as the sole carbon source and electron donor. Meanwhile, L-cysteine (0.2 mM), Na_2_S × 9H_2_O (0.2 mM), and DL-dithiothreitol (0.5 mM) were used as the reductants, while resazurin (1 mg/L) was used as the redox indicator [33]. The microcosms were incubated at 30 °C in the dark without agitation, and the chlorinated ethene concentrations in all enrichment cultures were monitored fortnightly. Each treatment was performed in triplicate. Various enrichment cultures with TCE-dechlorinating ability were successively transferred to freshly autoclaved solutions of the aforementioned media with 10% inoculum and were supplemented with 250-μM TCE and 10-mM lactate. Following repeated subculturing, we successfully developed three stable TCE-dechlorinating microcosms: LCS1 (enriched from the CS1 samples), LCS2 (enriched from the CS2 samples), and LAS (enriched from the AS samples). 

### 2.2. DNA Extraction, 16S rDNA Amplicon Sequencing

Approximate 4-g “original samples” (AS_0, CS1_0, and CS2_0) were collected directly from activated sludge/soil samples (AS, CS1, and CS2), and after repeated subculturing (about 18-months later), approximate 4-mL enrichment cultures from the “initial samples” after transferring into fresh medium (LAS_29, LCS1_18, and LCS2_18) and the “final samples” after TCE was depleted in the same batch incubation (LAS_31, LCS1_20, and LCS2_20), were filtered through 0.22-μm mixed cellulose ester membranes. Genomic DNA was extracted from all samples using the EZNA^®^ Soil DNA Kit (Omega Bio-Tek, Norcross, GA, USA), with the extraction and purification procedure performed according to the manufacturer’s instructions. The V4–V5 region of the prokaryotic 16S rRNA gene was amplified using the universal primers 515F (5′-GTG YCA GCM GCC GCG GTA-3′) and 907R (5′-CCG YCA ATT YMT TTR AGT TT-3′) [34]. PCR was performed under the following conditions: 95 °C for 3 min, 30 cycles of 95 °C for 45 s, 50 °C for 60 s, 72 °C for 90 s, and 72 °C for 10 min. Triplicate PCR products were combined and purified using the QIAquick PCR purification kit (Qiagen, Valencia, CA, USA). The 16S rDNA amplicons were sequenced on an Illumina MiSeq platform using a paired-end sequencing strategy (2 × 250 bp) at the Majorbio Bio-Pharm Technology Co., Ltd. (Shanghai, China). Since each treatment was performed in triplicate, 27 samples were sequenced. The 16S rDNA amplicon sequencing and the shotgun sequencing data were deposited in the National Center for Biotechnology Information’s short reads archive database under BioProject number PRJNA702557.

### 2.3. Analytical Methods and Data Analysis

Analyses of the chloroethenes and ethene were performed using the gas chromatography (GC, Agilent 7890A, CA, USA) equipped with a headspace sampler and a flame ionization detector. A 60-m × 0.32-mm DB-624 capillary column with a 1.8-μm film thickness (J&W Scientific Inc., Folsom, CA, USA) was used to separate the compounds. We withdrew 100-µL headspace samples using gastight 250-µL glass syringes (model 1725, Hamilton Co., Reno, NV, USA) and were manually injected into a split injector operated at a split ratio of 2:1. The conditions of the GC were as follows [12]: carrier gas = ultra-high-purity nitrogen, flow rate = 3.0 mL/min, injection temperature = 200 °C, and detector temperature = 220 °C, with the oven temperature programmed to hold at 50 °C for 3 min before increasing to 200 °C at a rate of 50 °C/min and finally holding at 200 °C for 2 min. The concentrations of chloroethenes and ethene were calculated by analyzing the headspace samples as previously described [31,35].

### 2.4. Quantification of RDase Genes 

In the quantification of the RDase genes (*tceA*, *vcrA,* and *bvcA*), qPCR reactions were performed in triplicate using an Applied Biosystems 7500 Real-time PCR system (Applied Biosystems, Waltham, MA, USA) alongside TaqMan primers and probe sets [36]. Each MicroAmp optical tube had a 20-μL reaction volume containing a 10-μL TaqMan Universal PCR Master Mix (Applied Biosystems), 0.4-μL forward primer and reverse primer (10 μM), and 0.8-μL TaqMan probe (300 nM each), 0.4-μL ROX Reference Dye; 6-μL Nuclease-Free Water and 2-μL DNA template from each 10-fold-diluted sample. PCR conditions were as follows: 30 s at 95 °C, followed by 40 cycles of 5 s at 95 °C and 34 s at 60 °C. The PCR was carried out in a spectrofluorimetric thermal cycler (ABI Prism 7700 Sequence Detection System, Applied Biosystems). The abundance of dehalogenase genes was assessed via the calibration curve according to a previously described method [33,36]. 

### 2.5. Sequencing Analysis 

The 16S rRNA gene amplicon reads were trimmed and processed using the LotuS pipeline tool [37]. The low-quality reads were excluded from further analysis [38], whereas the remaining high-quality reads were chimera-checked and clustered into operational taxonomic units (OTUs) at a 97% identity threshold using UPARSE [39]. The *α*- and *β*-diversity indices of the microbial communities were then processed after rarefaction to the same sampling depth using QIIME v1.9.0 [40]. Taxonomic annotation was performed using the RDP classifier and the SILVA database (v132) (https://www.arb-silva.de/ (accessed on 10 April 2018)), with a bootstrap cutoff of 80%.

### 2.6. Network Analysis

Networks were generated for OTUs grouped taxonomically at the OTU level, and used to identify the potential microbe–microbe interactions in anaerobic TCE-degrading consortia. The co-occurrence networks of the identified OTUs were constructed via the Spearman correlation and the Spearman’s rank coefficients (ρ) across all 97%-cutoff OTUs, with occurrence in 16 (out of 18) samples and were calculated pairwise using the R package Hmisc tool [41]. Subsequently, the significant (FDR-adjusted *p* value < 0.01) and robust (ρ ≥ 0.6) correlations among the OTUs and modular analysis were conducted by using the R package igraph tool [42]. Network visualization was performed using Gephi [43]. Both nodes with high betweenness centrality (BC, represented potential key connector or bottleneck species) and with high degree (represented hubs in the network) were indicators for potential keystone species [44]. 

### 2.7. Ecological Null Model Analysis

A null model approach, which is based on the weighted β-nearest taxon index (βNTI) in combination with Bray–Curtis based Raup-Crick (RC_bray_), was performed to quantify the contribution of the main ecological (deterministic or stochastic) processes underlying the assembly of taxonomic communities [45,46]. There are five ecological processes (homogenizing selection, variable selection, dispersal limitation, homogenizing dispersal, and undominated (ecological drift)) simulating by this null model [45,46]. The weighted β-mean nearest taxon distance (βMNTD) among the communities was calculated and imported to null model for testing the βNTI. The βNTI values are significantly higher (>2) and lower (<−2) than expected were considered as homogenizing and variable selection, respectively [47]. For the case of |βNTI| < 2, but with RC_bray_ values of >0.95 and <−0.95, the contributions were regarded as dispersal limitation and homogenizing dispersal, respectively. The trace contributions (|βNTI| < 2 and |RC_bray_| < 0.95) were identified as undominated processes. 

### 2.8. Statistical Analysis

In order to assess the enriching effect by lactate-feeding and TCE-supplementing, and explore the microbial community succession patterns during the enriching process, the indicator OTUs for these communities were classified using IndVal analysis with the R package labdsv tool [48]. Only OTUs with highly significant indicator values (IndVal index >0.95, *p* < 0.001) were considered strict habitat specialists [49]. Principal coordinate analysis (PCoA) was used to visualize the *β*-diversity pattern of the microbial communities based on Bray–Curtis distance matrices. In addition, permutational multivariate analysis of variance (Adonis) and analysis of similarity (ANOSIM) were used to test the significant variations in the microbial communities among the different enrichment cultures (LAS, LCS1, and LCS2) [21,50]. Statistical analyses and plots were performed using the R packages ComplexHeatmap [51], ggplot2 [52], phyloseq [53], and vegan [54] tools.

## 3. Results

### 3.1. Dechlorination of TCE by Enrichment Culture

**Figure 1:**
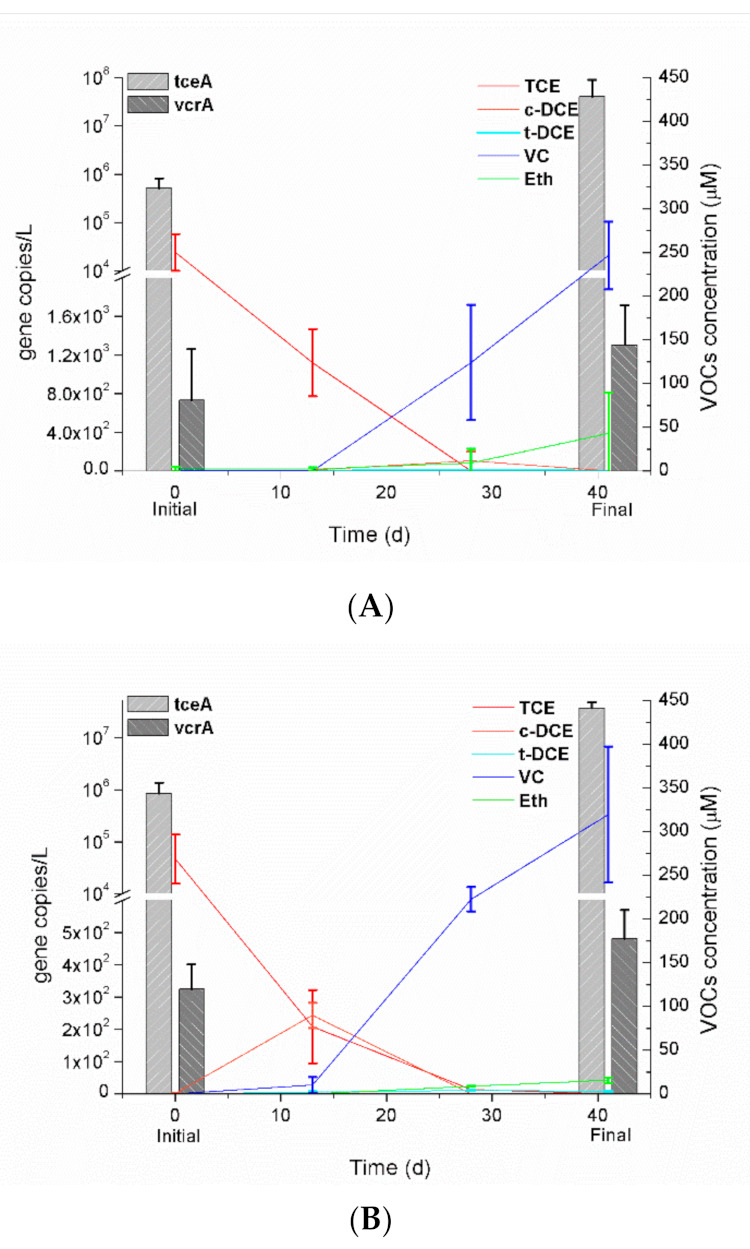

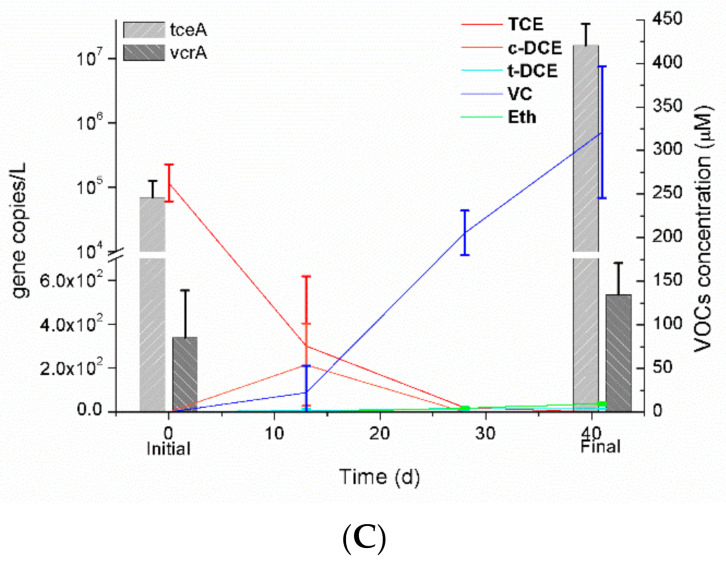
Reductive dechlorination of TCE during the incubating period in microcosms LAS (**A**) established with activated sludge in Xiamen, and LCS1 (**B**) and LCS2 (**C**) from chlorinated solvent-contaminated soil in Shanghai. Each microcosm was supplied with 250 µM of TCE and 10 mM of lactate dissolved in anaerobic medium. Copy numbers determined by qPCR of *tceA* and *vcrA* in each microcosm were shown above each set of columns. Concentration of VOCs (including TCE, c-DCE, t-DCE, VC and Eth) in each microcosm were shown above each set of curves. Data were averaged from triplicate microcosms.

Mixed cultures capable of dechlorinating TCE were successfully enriched from CS and AS samples with lactate as the sole carbon source and electron donor. The chlorinated solvents and ethene concentration trends of dechlorinating cultures with fresh inoculum were then monitored. Figure 1 presents the variations in TCE, cis-DCE (cDCE), tDCE, VC, and Eth concentrations in each microcosm following inoculation. On transferring the samples to a fresh medium, TCE dechlorination to cDCE and tDCE was evident in each microcosm, and with this transformation completed in 28 days and 41 days in the LCS and LAS microcosms, respectively. The daughter product, cDCE was converted into VC continually during treatment, while the tDCE appeared to remain at a low concentration (≤4 μM). The VC was subsequently dechlorinated very slowly to ethene, which was detected in 14 days following inoculation, with the concentration gradually increasing over time. In the meanwhile, three previously identified DHC RDase genes (*tceA*, *vcrA*, and *bvcA*) were confirmed via PCR with primer sets [36] prior to quantification. All enrichment consortia produced strong PCR products for both *tceA* and *vcrA*, whereas the detection of *bvcA* did not yield a positive signal, indicating the presence of *tceA*- and *vcrA*-carrying dechlorinators but the absence of *bvcA*-carrying dechlorinators. The quantification results (Figure 1) showed that the *vcrA* gene copy numbers were relatively stable at a low level, albeit slightly increased over time. Simultaneously, the *tceA* abundances all significantly increased from a 10^5^ to a 10^7^ level, indicating that the overall dechlorination ability of the enrichment cultures could be due to the combined activities of *tceA*- and *vcrA*-carrying dechlorinators, with higher dechlorinating activity of TCE to DCEs to VC, but lower dechlorinating activity of VC to Eth.

### 3.2. Variation in Microbial Community Composition of Enrichment Cultures

**Figure 2:**
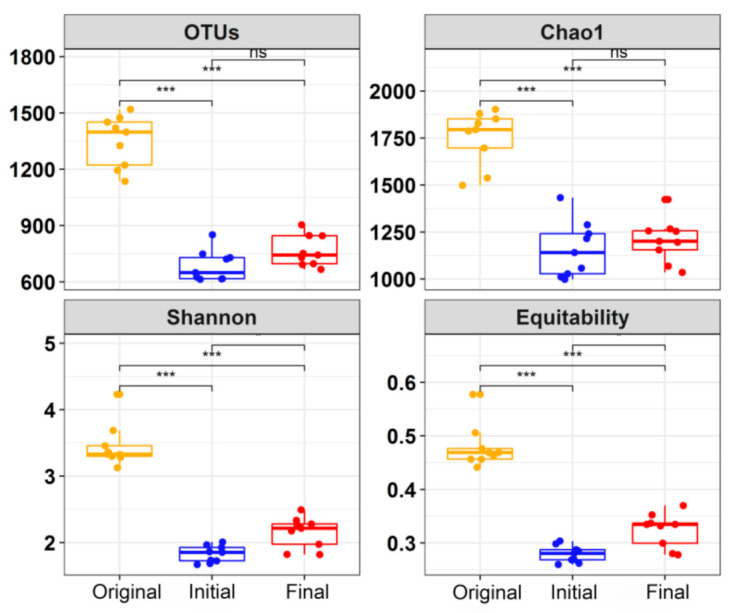
Comparison of *α*-diversity indices of the prokaryotic communities in all enriching samples at different time points: Original (9 samples), Initial (9 samples) and Final (9 samples). Error bars indicate standard deviation of the mean, and the asterisks indicate significant difference (*** *p* < 0.001), whereas “NS” indicates no significant differences.

High-quality reads (147,212–283,139) representing 3263 OTUs were generated through the quality filtering of a total of 5,671,663 reads across the 9 original and 18 enriched samples. The results of the *α*-diversity analysis indicated that the phylogenetic diversity (Shannon diversity and equitability) and richness (OTUs and Chao1 richness) were significantly higher in the original samples than those in the enriched samples (including initial and final samples) communities (Wilcoxon test, *p* < 0.001) (Figure 2). These results imply that the microbial communities in the original samples were more diverse than those in the enriched samples. In addition, a slightly higher *α*-diversity was observed for the microbial communities of the final samples than those of the initial samples in the same batch (Figure 2). 

**Figure 3:**
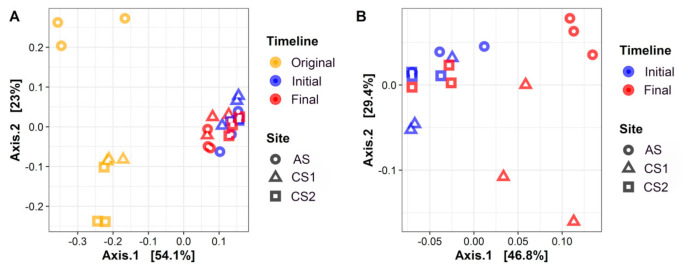
PCoA ordination analyses based on Bray–Curtis dissimilarity matrix of the prokaryotic communities in three microcosms (enriched from CS1, CS2 and AS samples) over the (**A**) whole timeline (Original, Initial and Final) and (**B**) incubation timeline (Initial and Final).

PCoA ordination indicated that the enriched samples were clustered more tightly than the original samples (Figure 3A), and that communities of the original and final samples from each site were clearly separated (Figure 3A,B), but communities of the initial samples were clustered together (Figure 3B). The communities of the AS and CS1 samples were separated from each other over the sampling time (Figure 3A,B), but the initial and final samples originating from the CS2 site were clustered together (Figure 3B). The Adonis and ANOSIM analyses further confirmed that the compositions of the whole communities were significantly different between the original samples and enriched samples (Adonis: R^2^ = 0.535, *p* < 0.001, ANOSIM: R = 0.504, *p* < 0.001), as well as among the samples originating from different sites (Adonis: R^2^ = 0.316, *p* < 0.001, ANOSIM: R = 0.263, *p* < 0.01) (Table 1). Moreover, the microbial taxonomic communities in the original samples had greater *β*-diversity than those in the enriched samples (Kruskal–Wallis test, *p* < 0.001), and they were also greater in the final samples than in the initial samples (Kruskal–Wallis test, *p* < 0.001) (Figure S1).

The heatmap result of the shared OTUs also presented the similar distribution patterns with the PCoA analysis. The original, initial and final samples were discerned clearly except for the LCS2_20 and LAS_29_2 samples (Figure S2). Further analysis found that the enriched samples (including initial and final samples) shared more OTUs than the original samples, which indicated that there would be convergence in the microbial community structure after a long-term enriching period under the same conditions. 

### 3.3. The Ecological Processes Underlying the Assembly of Enriched Microbiomes

**Figure 4:**
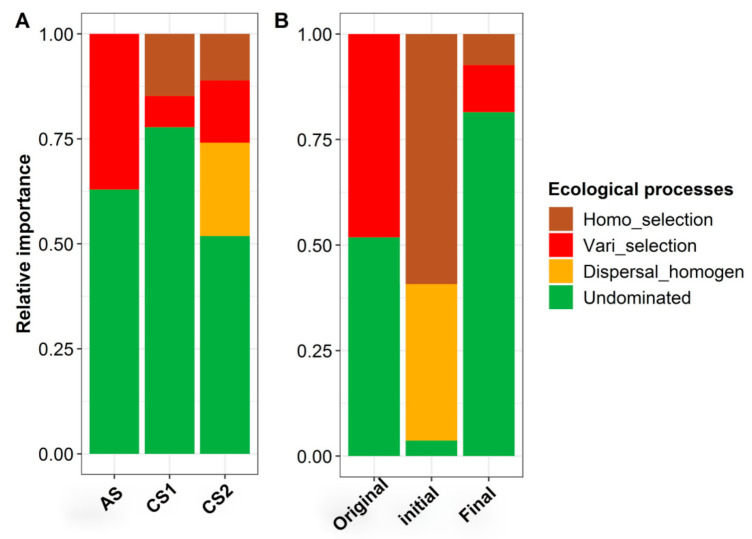
The contributions of ecological processes to microbial assemblages in each of the enriching samples originated from different sites (**A**) at different time points (**B**). Weighted *β*-nearest taxon index (*β*NTI) were applied to evaluate the assembly processes, where the deterministic processes mainly included homogeneous and variable selections, and the stochastic processes included dispersal limitation, homogenizing dispersal, and undominated processes.

The ecological processes underlying the structure of the microbial communities were deciphered using a quantitative ecological framework, which incorporated the phylogenetic (*β*NTI) and taxonomic (RC_bray_) *β*-diversity dissimilarity-matrix-based null models. The results indicated that an undominated process was the main process in determining the assembly of the microbial communities within the microbiomes of AS, CS1, and CS2 (51.9–77.78%), while variable selection (7.4–37.0%) played a certain role in these microbiomes. Here, homogenizing selection (11.1% and 14.8%) shifted the ecological assembly processes of the CS1 and CS2 microbiomes to a certain extent, whereas homogenizing dispersal (22.2%) played a relatively important role in shaping the CS2 communities (Figure 4A). Along the enriching timeline, an undominated process (51.9%) and variable selection (48.1%) were the main processes governing the turnover of the original sample communities, whereas homogenizing selection (59.3%) and homogenizing dispersal (37.0%) were the most important processes in determining the assembly of the microbial communities at the initial stage, with an undominated process (81.5%) playing the key role, alongside limited contributions from homogenizing selection (7.40%) and variable selection (11.1%), in shaping the communities of the final time samples (Figure 4B).

### 3.4. Distribution of Indicators during the Enrichment Process

**Figure 5:**
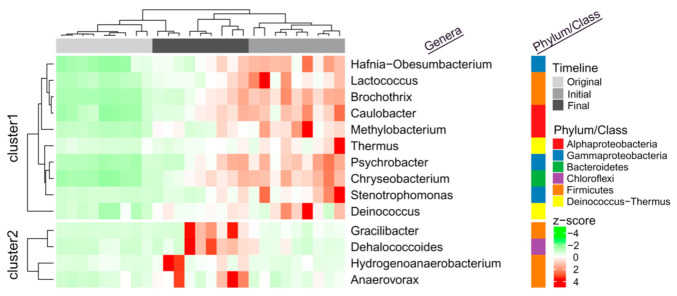
Heatmap diagram showing the distribution of 14 prokaryotic indicators (genera level) during the enriching and incubating period. Each row and column of the heatmap diagram corresponds to a single indicator and samples, respectively. The row data for each indicator were z-score transformed. Dendrograms were constructed based on Pearson correlation clustering. The grey and dark grey colors in the column annotations indicate the Original, Initial and Final samples, respectively. The row annotations on the right-hand side indicate the phylum/class of each indicator.

Homogenizing selection, as a deterministic force, drives the turnover of the microbial communities during the incubating period, which exhibited distinct and stage-specific characteristics. Indicator species analysis showed that 10 genera (*Hafnia-Obesumbacterium*, *Lactococcus*, *Brochothrix*, *Caulobacter*, *Methylobacterium*, *Thermus*, *Psychrobacter*, *Chryseobacterium*, *Stenotrophomonas,* and *Deinococcus*) and genera (*Gracilibacter*, DHC, *Hydrogenoanaerobacterium,* and *Anaerovorax*) were highly associated with the microbial communities at the initial and final stage samples, respectively (Figure 5). However, no indicators were observed at the original stage samples (Figure 5) contributing to the stochastic force, which shaped the primary community composition to become more complex and homogeneous.

### 3.5. Topological and Taxonomic Properties of the Co-Occurrence Networks

We used co-occurrence networks to evaluate any potential microbial interactions and to identify keystone species that are especially interactive. The entire network derived from the TCE-dechlorinating enrichment microbiome dataset (including 18 samples without the original samples), which consisted of 168 nodes with 229 edges. 

**Figure 6:**
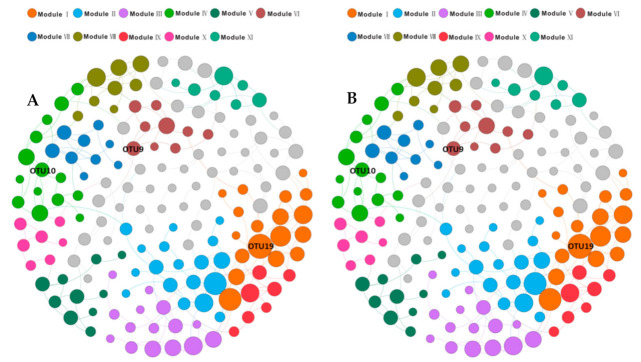
Networks of TCE-dechlorinating cultures microbiome. Co-occurrence networks of 97%-cutoff operational taxonomic units (OTUs) within TCE-dechlorinating cultures microbiome based on correlation analysis. The size of each node is proportional to the number of connections (i.e., degree). The edges indicate strong Pearson Correlation Coefficient (r > 0.77) and significant (*p* < 0.05) positive correlations between nodes. (**A**): OTUs colored by modularity class; (**B**): OTUs colored by the phyla/classes-level taxonomy.

Based on their connectivity pattern, we clustered the OTUs using modularity maximization, resulting in 11 major modules, each of which was composed of a group of OTU nodes that interconnected more frequently among themselves than with nodes in other modules (Figure 6A, Table S1). The modules could be perceived as functional units in the microbial communities, whereas previous studies have also interpreted the modules as niches [55]. Among them, *Pseudomonas* dominated almost all of module I, II, and III, and was highly connected to other species within and outwith Module I; while *Desulforhabdus* (OTU_9) with the highest BC value, was highly connected to other species in and outside of its own Module VI. As the predominant TCE dechlorinator, the genus DHC (OTU_10) was highly connected to *Christensenellaceae*, *Gracilibacteraceae*, and an unknown *Clostridiales* bacterium in its own Module IV, which was regarded as the potential dechlorinating center. Most notably in this network, the genus *Methanofollis* (OTU_4), as the sole identified archaea group, was assigned to Module VIII, which might play a special role in the dechlorination process.

**Figure 7:**
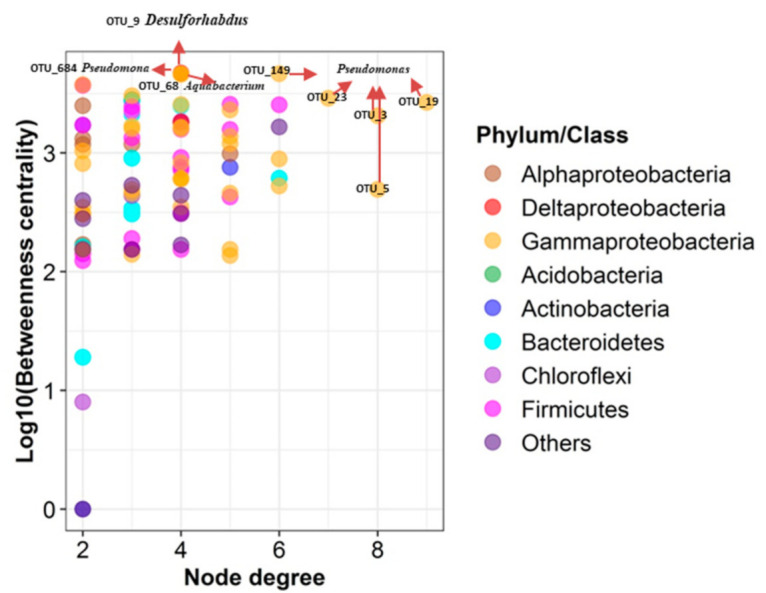
Keystone species analysis. Betweenness centrality vs. node degree of all species in the prokaryotic community networks of TCE-dechlorinating microcosms. Nodes with high betweenness centrality represented potential key connector (or bottleneck) species. Nodes with high degree represented hubs in the network. Both measures were indicators for potential keystone species. Bacterial species (dots) were colored by phylum/class membership. Bacterial species that were maximal in property are highlighted in plots. The genus *Pseudomonas* and *Desulforhabdus* may act as potential keystones in the dechlorinating microbiome.

Based on their evolutionary relationships, all OTUs in the network were classified into seven major phylum/classes: *Gammaproteobacteria* (39.3%), *Firmicutes* (16.7%), *Alphaproteobacteria* (9.52%), *Actinobacteria* (8.33%), *Bacteroidetes* (7.74%), *Chloroflexi* (5.36%), and *Deltaproteobacteria* (2.38%) (Figure 6B, Table S1). The genera (including *Pseudomonas*, an unknown genus in the *Clostridiales* family XII, *Aminivibrio*, *Psychrobacter*, *Flavobacterium*, *Acinetobacter* with a higher degree value, in company with the genera (including *Desulforhabdus*, *Pseudomonas*, *Aquabacterium*, *Acetobacterium*, an uncultured genus in *Anaerolineaceae*, *Lysobacter*, an uncultured genus in *Saprospiraceae* (OTU_59), and an unknown genus in *Holophagae* subgroup 7 with a higher BC value were regarded as the potential keystone species (Figure 7, Table S2).

## 4. Discussion

### 4.1. Performance of the Reductive Dechlorination of TCE by Enriched Cultures

In this study, we successfully enriched three stable TCE-dechlorinating consortia using lactate as the sole carbon source and electron donor. Since chlorinated ethene respiring microorganisms may exist in sites that have not been reported to have chlorinated solvent contamination [56,57,58,59], it is not surprising that TCE dechlorination occurred in our enriched cultures, which were either from chlorinated solvent-contaminated sites or from activated sludge. All enriched cultures were able to completely remove 250-μM TCE in 28–41 days, achieving the TCE-degradation rate of 6.1–8.9 μM/day (Figure 1), which proceeded more slowly than those observed in previous studies (with the TCE-degradation rate of 11–50 μM/day) involving lactate-enriched cultures under similar conditions [26,27,28]. Furthermore, partially reductive dechlorination of TCE was observed in our consortia, with a buildup of VC and a limited generation of Eth, which indicated the possible inhibition of VC dechlorinators. DHC is the only known genus that can reduce VC to Eth [29], and the existence and abundance of DHC in our enriched consortia were confirmed via qPCR analysis of RDase genes and 16S rRNA gene amplicon sequencing. In fact, DHC was determined to be an indicator in our enriched consortia at the final time of the incubation period (Figure 5), as well as the key dechlorinator that responds most positively to the TCE-dechlorinating process (Figure 1). The achievement of complete reductive dechlorination is strictly dependent on the key microorganisms equipped with the RDase enzymes, among which are the *tceA* gene coding for a protein that catalyzes the sequential metabolic transformation of TCE to cDCE and VC, and the cometabolic generation of Eth from VC. Meanwhile, *vcrA* reduces DCE isomers and VC, while *bvcA* is involved in the metabolic transformation of VC to Eth [60]. In our study, the *tceA* gene was detected with abundance and significantly increased, which indicated that the dechlorination of TCE to DCE and VC may be dominated by the *tceA*-carrying populations and the stable low level of the *vcrA* gene could explain the accumulation of VC and the presence of a limited amount of Eth. Reductive dechlorinations catalyzed by dechlorinators produce final products that vary depending on the physiological groups involved and may occur via a combination of metabolic and cometabolic processes [26]. The accumulation of VC has also been frequently observed in various field and laboratory experiments since VC reduction is the rate-limiting step in the reductive dechlorination of TCE to Eth [61]. As is well known, VC is a human carcinogen and is more toxic than its parent compounds. Appropriate supplementation with substrates promoting complete dechlorination is the key element to determine the success of the biostimulation approach.

### 4.2. Interactions of TCE Dechlorinators and Other Keystone Species in the Enriched Consortia

Lactate can be used as a common growth substrate for major groups of bacteria, which could catalyze lactate to pyruvate via lactate dehydrogenase [62], including *Pseudomonas* spp. [63,64], *Acinetobacter* spp. [65], sulfate-reducing bacteria (SRB), and acetogenic bacteria [62]. The microbial community of enrichment cultures is complex, the analysis of co-occurrence networks using abundant data could enable a better understanding of microbial community ecology through identifying the interactome relationships among species that cannot be obtained through the use of traditional analytical approaches [66,67]. From the analysis results for the microbial interactome networks and the potential syntrophic relationships in our enriched cultures, we ascertained that *Pseudomonas* populations with the highest node degree and highest proportion (>74% of the classified sequences) were present in our dechlorinating consortia. *Pseudomonas* spp. has been reported as a dechlorination bacteria [68,69,70], and might play an indirect but crucial role in the dechlorinating process by merging into the pyruvate cycle, which operates the energy production and regulates the TCA cycle. Another identified dominant group contained SRB, including *Desulforhabdus* spp. (accounting for 0.05–0.16% of the classified sequences) with the highest BC (Figure 6) and *Desulfovibrio* spp. (accounting for 0.35–10.03% of the classified sequences), which were also identified as keystone members that can utilize lactate as the preferred electron donor [71]. Syntrophic lactate oxidation in the presence of a hydrogenotrophic methane-producing partner has been described in relation to *Desulfovibrio* spp. [72], which belongs to incomplete oxidizing SRB and could not use acetate as an electronic donor [73]. When involving in the anaerobic dechlorination of TCE, *Desulfovibrio* spp. plays a role in scavenging O_2_ and providing necessary nutrients [74]. Meanwhile, *Desulforhabdus* spp. could oxidize formate, acetate, propionate, and ethanol during sulfate reduction [73], competing with DHC in terms of acetate. As a member of hydrogenotrophic methanogens [72], *Methanofollis* spp. (accounting for 0.02–7.92% of the classified sequences) was the sole archaea member detected in our enriched consortia, which would compete with DHC in terms of H_2_. 

DHC is strictly hydrogenotrophic and is fastidious regarding its growth conditions, including pH, H_2_, acetate, corrinoids, biotin, and thiamine, all of which are generally provided by coexisting members of anaerobic food webs within dechlorinating communities [19,75]. To survive and to undergo complete dechlorination to Eth, in addition to the collaboration with other microorganisms, DHC also must compete for nutrient substances and H_2_ with other members. The limited generation of Eth and the accumulation of VC identified in our study indicate that the current environment does not provide favorable conditions for complete TCE dechlorination. While reductive dechlorination can occur under a variety of redox conditions, the reaction commonly only accounts for a small fraction of electron flow in microbial communities, with other terminal-electron-accepting processes, such as sulfate reduction, iron reduction, nitrate reduction, methanogenesis, homoacetogenesis, and volatile fatty acid formation typically accounting for a large fraction of the electron flow in these systems [76]. While DHC may play the dominant role in TCE-dechlorinating processes, it was clearly not the predominant member (only accounting for 0.05–2.03%) in our enriched culture. Given that the microbial fermentation of lactate proceeds rapidly and generates a high concentration of H_2_, a considerable amount of H_2_ is consumed during alternate terminal electron-accepting processes, thus limiting the efficiency of reducing the equivalent consumption in the reductive dechlorination process [12]. How to shape the lactate-feeding community for competent TCE dechlorination thus requires further investigation.

### 4.3. Assembling Mechanism of Stable TCE-Dechlorinating Communities

Lactate is considered as one of the favorable feedstock for major groups of bacteria and is also a key intermediate in the anaerobic digestion of biomass in the environment [72]. In our study, long-term feeding with lactate and TCE contributed to a stable consortium capable of TCE dechlorination. Revealing the ecological processes driving the assembly of the aforementioned TCE-dechlorinating communities is of significance to the bioremediation of TCE-contaminated sites. Here, we found that the communities of our enriched cultures were shaped by both stochastic and deterministic processes (Figure 4A). A recent study proposed that environmental heterogeneity and regional processes were the primary factors to determine the community structure [77]; hence, variable selection played an essential role in the formation of the unique microbial assemblages in the original samples (Figure 4B), and greater *β*-diversity (Figure S1) due to distinct primary environment was observed among the different original samples. 

When the samples were transferred into the same enrichment medium, their community patterns began to converge (Figure S1) under the same environment with repression and induction of the lactate-TCE-feeding. For the alteration of environmental conditions, certain microorganisms may become locally extinct or may be maintained at low levels, while they may also become more abundant and capable of playing a greater role in the given ecosystem [78]. For the initial stage samples, homogenizing selection process drove the alteration of the community structure (Figure 4B), resulting in the decrease in *α*-diversity (Figure 2). Meanwhile, homogenizing dispersal also played a certain role (Figure 2) for the operation of transferring to different serum bottles. Subculturing procedure might bring a trace amount of oxygen, which will contribute to the emergence of facultative anaerobic populations such as *Hafnia-Obesumbacterium* [79], *Psychrobacter* [80], *Chryseobacterium* [81,82], and *Stenotrophomonas* [83]. Simultaneously, supplementation with abundant lactate can increase the number of lactate-metabolizing groups (LMG) represented by the genera of, for example, *Pseudomonas*, *Lactococcus*, *Brochothrix*, *Thermus*, and *Deinococcus* (Figure 5). 

With the generation of secondary metabolites and the reduction in TCE toxicity, enrichment communities adapt to the culturing environment (such as carbon source, pH, content of pollutants, anaerobic degree), and the stochastic process revert back to dominate the turnover of microbial community (Figure 4). In the final stage samples, the recovery of *α*-diversity was observed in all enrichment cultures (Figure 2). Lactate utilization may provide sufficient direct electron donors and related cofactors to activate the strictly anaerobic and/or dechlorinating-related populations, such as DHC, *Gracilibacter*, *Hydrogenoanaerobacterium,* and *Anaerovorax* (Figure 5). However, the stochastic process will increase the phylogenetic diversity (Figure 2) and develop a more complex community structure (Figure S1), which was not beneficial for the accumulation of DHC.

The aforementioned assembling mechanism indicates that successive stimulation with appropriate substrates can promote bioremediation for TCE-contamination, and also hints to us that the reduction of the stochastic forces or the enhancement of the deterministic processes may promote more effective biostimulation.

## 5. Conclusions

In summary, this study revealed the succession pattern and associated assembly mechanisms of lactate-fed consortia for the reductive dechlorination of TCE. The long-term subculturing results for the microbial communities of the enrichment cultures changed along with time, while the different sample sources tended to shape distinct patterns of the microbial community, and the indicator pattern also exhibited distinct and enrichment time-specific characteristics. The co-occurrence network showed that complete TCE dechlorination in our enrichment cultures required cooperation by keystone populations such as DHC, LMG, SRB. While repeated subculturing enhanced the selecting effect, deterministic and stochastic processes were simultaneously responsible for shaping the TCE-dechlorinating community assembly. The achievement of stable TCE-dechlorinating consortia presents an effective strategy for the bioremediation of TCE-contaminated sites.

## Figures and Tables

**Table 1:** Significance test of the structure of the prokaryotic communities between sample sources and enriching time using Adonis and ANOSIM analysis.

	Factor ^b^	Adonis		ANOSIM	
		R^2^	*p*	R	*p*
Whole ^a^	Site	0.082	<0.01	0.028	0.214
	Time	0.535	<0.001	0.504	<0.001
	S * T	0.228	<0.001	—	—
Incubation	Site	0.316	<0.001	0.263	<0.01
	Time	0.223	<0.001	0.235	<0.01
	S * T	0.148	<0.05	—	—

^a^ Whole, the prokaryotic communities during the enriching period (from original to initial time) and incubating period (from initial to final time); incubation, the prokaryotic communities during the incubating period. ^b^ Site, sites of sample sources, including contaminated soil (CS1 and CS2) and activated sludge (AS); time, sampling time, including three time points (original, initial and final); S * T, the interactive effects of sample sources and time.

## Supplementary Materials

The following are available online at https://www.mdpi.com/article/10.3390/microorganisms9040751/s1, Figure S1: Box plots of the variance in the Bray–Curtis distance of taxonomic communities in different sampling time point; Figure S2: Heatmap showing the percentage of OTUs shared between any two samples in this study; Table S1: Topological attributes of modules and taxonomies information in the co-occurrence networks of microbial communities; Table S2: Topological attributes of nodes and taxonomies information in the co-occurrence networks of microbial communities.
