# Supplementary material for: Characterization and Performance of Lactate-Feeding Consortia for Reductive Dechlorination of Trichloroethene
Source: Microorganisms. 2021 Apr 2;9(4):751. doi: 10.3390/microorganisms9040751 (PMC8065584; doi:10.3390/microorganisms9040751)

microorganisms

MDPI

Supplemental Materials

Characterization and performance of lactate-feeding consortia for reductive dechlorination of trichloroethene

Jiangwei Li [1,2], Anyi Hu [1], Shijie Bai [3], Xiaoyong Yang [1], Qian Sun [1], Xu Liao [1], Chang-Ping Yu [1,4*]

1 CAS Key Laboratory of Urban Pollutant Conversion, Fujian Key Laboratory of Watershed Ecology, Institute of Urban Environment, Chinese Academy of Sciences, Xiamen 361021, China; jwli@iue.ac.cn (J.L.); ayhu@iue.ac.cn (A.H.); xyan@env.dtu.dk (X.Y.); qsun@iue.ac.cn (Q.S.); xliao@iue.ac.cn (X.L.); cpyu@iue.ac.cn (C.-P.Y.);
2 University of Chinese Academy of Sciences, Beijing 100049, China;
3 Institute of Deep Sea Science and Engineering, Chinese Academic of Sciences, Sanya 572000, China; baishijie@idsse.ac.cn (S.B.)
4 Water Innovation, Low Carbon and Environmental Sustainability Research Center, National Taiwan University, Taipei 10617, Taiwan;
* Correspondence: cpyu@iue.ac.cn (C.-P.Y.)

Table S1. Topological attributes of modules and taxonomies information in the co-occurrence networks of microbial communities. Please find table S1 in the supplement excel file.

Table S2. Topological attributes of nodes and taxonomies information in the co-occurrence networks of microbial communities. Please find table S2 in the supplement excel file.

Figure S1. Box plots of the variance in the the Bray-Curtis distance of taxonomic communities (Kruskal-Wallis test, $P < 0.01$) in different time point (i.e.,Original, Initial and Final). The asterisks indicate significant difference (***$P < 0.001$).

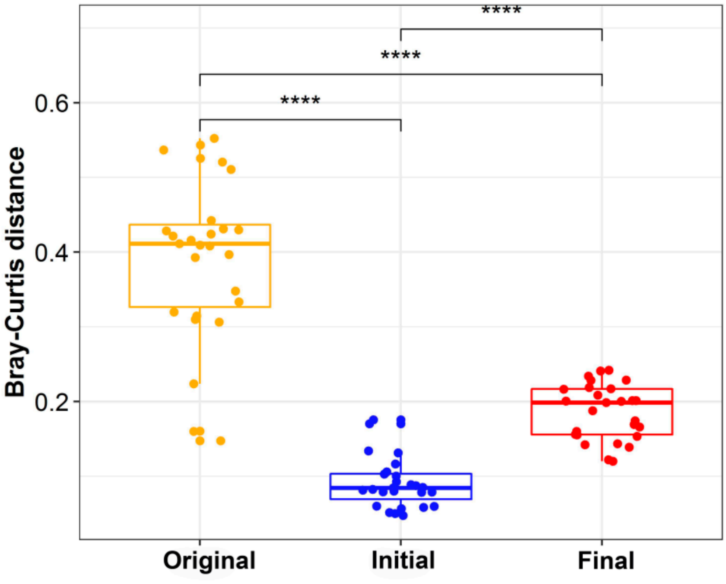

Figure S2. Heatmap showing the percentage of OTUs shared between any two samples in this study. Community data were also clustered to better reveal the structure in the dataset. Sample names indicate enriching carbon sauce, original sample sauce, enriching time, as well as triplicates number. The grey and dark grey colors in the column annotations indicate the Original, Initial and Final samples, respectively. Red and green colors symbolize higher and lower relative abundances, respectively.

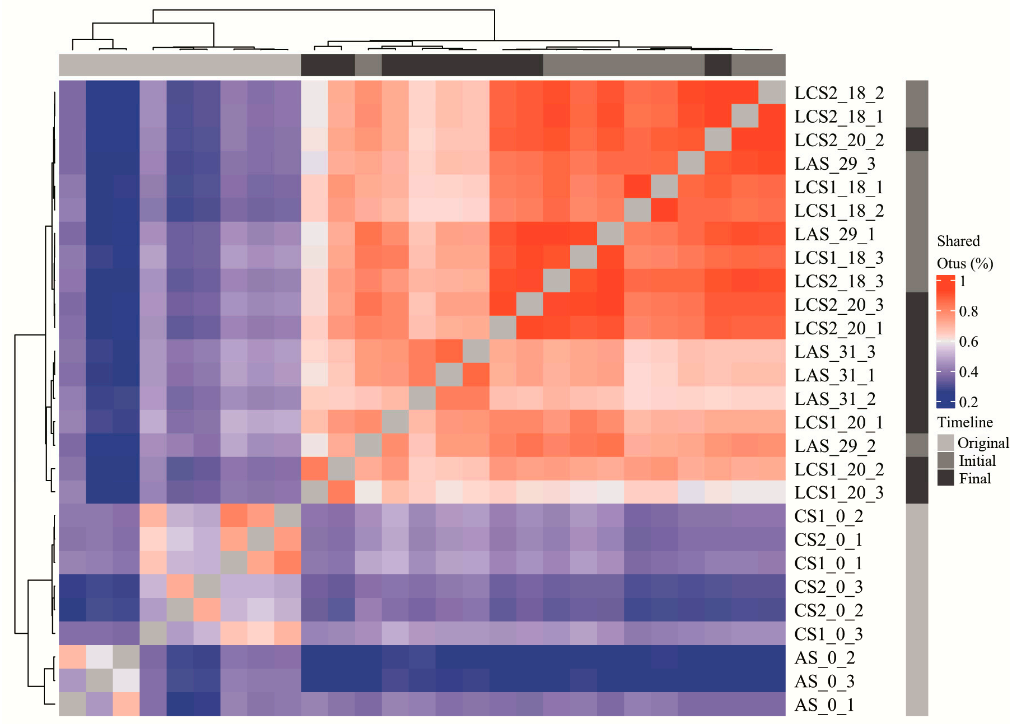

Supplement: Supplementary file 1 [file microorganisms-09-00751-s001.zip › Supplementary Files_TCE-dechlorination.pdf]
